# The institutional afterlife of a pandemic: Zimbabwe’s One Health strategy and the limits of crisis-driven governance

**DOI:** 10.1080/16549716.2026.2761182

**Published:** 2026-11-11

**Authors:** Cliff Zinyemba

**Affiliations:** Centre on Climate Change and Planetary Health, Medical Research Council Unit the Gambia at London School of Hygiene and Tropical Medicine, Banjul, The Gambia; Department of Global Public Health and Family Medicine, Faculty of Medicine and Health Sciences, University of Zimbabwe, Harare, Zimbabwe; The Health Research Unit Zimbabwe, Biomedical Research and Training Institute, Harare, Zimbabwe

**Keywords:** Health system resilience, health financing, multisectoral coordination, policy implementation, Southern Africa

## Abstract

Governance structures built during a crisis often outlive the emergency that produced them. Zimbabwe’s National One Health Strategic Plan 2026–2030 (the Plan) is a direct institutional legacy of the COVID-19 pandemic. Its coordination structure sits in the Office of the President and Cabinet rather than a line ministry, deliberately echoing the Whole-of-Government arrangement credited with the country’s pandemic response. This debate piece asks whether governance built in a crisis survives once the crisis is over. It draws on the Plan itself, a 2024 baseline assessment of One Health in Zimbabwe, the self-assessment phase of the country’s 2025 Joint External Evaluation of health security capacities, and published case studies from Kenya and Rwanda. I argue that the pandemic generated genuine political priority for One Health, but that institutionalisation has not kept pace. The Plan is costed in full, yet it carries no earmarked domestic allocation, no instrument establishing its coordination mechanism in law, and no reference to provincial or district implementation. Zimbabwe is best read as an illustrative early case rather than as evidence of a regional trajectory. As many governments turn pandemic-era emergency coordination into permanent health structures, it offers an early test of whether such institutions can outlast the memory that built them or quietly fade once political attention moves on.

## Paper Context

- **Main findings**: Zimbabwe’s presidentially anchored One Health Strategic Plan reflects real political commitment generated by the coronavirus pandemic, but the Plan is costed in full without an earmarked domestic allocation, no legal instrument establishes its coordination mechanism, and its text does not refer to provincial or district implementation.
- **Added knowledge**: Crisis-driven political priority and institutional durability are separate processes that need not arrive together, and Zimbabwe is an illustrative early case as more governments make pandemic-era coordination bodies permanent.
- **Global health impact for policy and action**: Governments elevating health coordination after a crisis should treat financing, legislation and subnational implementation as deliberate, separate commitments, and should not assume that political visibility will secure them.

## Background

The organisational structures, emergency powers and coordination habits a crisis produces tend to persist after the acute phase and shape governance long after the emergency has faded from memory. Zimbabwe’s National One Health Strategic Plan 2026–2030 (hereafter, the Plan), launched in late 2025, puts that pattern to the test. The Plan places One Health coordination, spanning human, animal, plant and environmental health, in the Office of the President and Cabinet instead of a line ministry, to strengthen coordination ‘from the highest level’ [1]. The President’s foreword makes clear that this was deliberate, stating that the Plan draws on ‘best practices and lessons learnt from the very successful Whole of Government and Society national COVID-19 pandemic response, that my Office led and coordinated’ [1]. This is a Current Debate piece reporting no new data. Its three dimensions, financing, legal institutionalisation and subnational implementation, come from the Plan’s own strategic pillars and two assessments of One Health in Zimbabwe, not from an external framework.

## Political priority versus institutionalisation

More governments are turning pandemic-era coordination bodies into standing institutions, so the question matters well beyond Zimbabwe. Shiffman and Smith show that political priority is often driven by strong leadership and effective framing during a crisis, does not on its own guarantee institutionalisation, and frequently weakens once the individuals or circumstances that generated it change [2]. Public-policy scholarship reaches a similar conclusion by a different route, finding crises neither necessary nor sufficient for institutional reform, which depends instead on context, sustained leadership, resources and political support [3]. Walt and Gilson noted that a policy’s content can be strong even where the political process and institutional context around its implementation are weak [4].

A continental precedent comes from the Africa Centres for Disease Control and Prevention, established by the African Union in 2017, largely in response to the coordination failures exposed by the 2014–2016 West African Ebola epidemic [5]. Its growth since shows that crisis-born institutions can endure where political and financial investment continues. This piece asks whether Zimbabwe’s Plan has secured that follow-through on financing, legal mandate and subnational reach (Figure 1).

**Figure 1.**
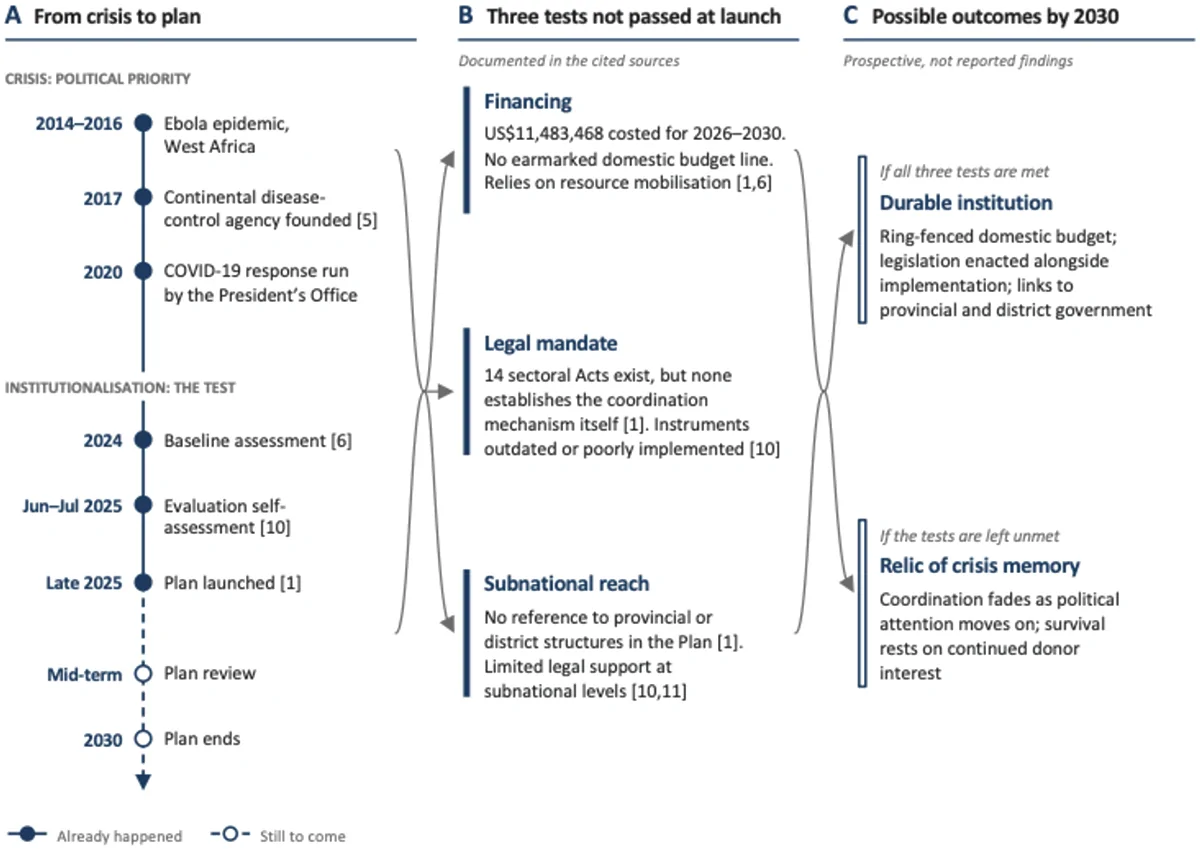
Zimbabwe’s national One Health Strategic Plan 2026–2030 as a test of crisis-driven governance. (A) Crises that generated political priority and the Plan’s path to launch. (B) The three institutionalisation tests unmet at launch. (C) Outcomes the Plan could reach by 2030, the author’s prospective interpretation. Filled markers denote what has happened; open markers what is to come. Sources [1,5–8].

## Presidential anchorage as an enabling condition

A 2024 baseline assessment of Zimbabwe’s One Health landscape found that, before the Plan, governance was focused largely on antimicrobial resistance, with ‘minimal coordination of initiatives across other sectors’ [6]. It combined a desktop review, focus group discussions and key informant interviews with senior experts across government, academia, parastatals and private organisations, validating its findings through net-mapping and strengths–weaknesses–opportunities–threats workshops [6]. Its authors concluded that there was ‘potential to place [One Health] governance above the ministerial level to improve coordination’ [6]. Presidential anchorage addresses that gap, giving the coordinating structure a convening authority no line ministry could command alone. It is best understood as a potentially enabling arrangement rather than a sufficient condition, since convening authority determines who comes to the table, not whether those parties hold the budgets, legal powers and subnational structures needed to act.

Zimbabwe is not alone. A continent-wide scoping review found presidential task forces and multisectoral response teams to be a common response to the Ebola and COVID-19 emergencies across Africa, frequently without the investment needed to turn them into lasting systems [9]. Kenya and Rwanda serve as comparators because peer-reviewed assessments of their mechanisms address where each sits in the executive, and because the Rwandan and Zimbabwean assessments came out of the same regional baseline exercise [6,10]. Kenya institutionalised One Health in 2011 through a Zoonotic Disease Unit jointly established by the human and animal health ministries, while antimicrobial resistance came to be coordinated through a separate interagency committee [11]. It now runs four topic-specific mechanisms, and its evaluators identify the absence of any structure linking them as the main gap [11]. Rwanda, among the few African countries with a standing One Health policy, has published evidence recommending that it relocate its coordinating unit to the Prime Minister’s Office [10]. Both are recommendations for future reform rather than demonstrated outcomes, though they show a regional inclination to move weak cross-sectoral coordination higher up the executive, a remedy Zimbabwe applied from the outset.

## Documented gaps in institutionalisation

Two separate assessments, the 2024 baseline study [6] and the self-assessment phase of the 2025 Joint External Evaluation [7], agree that political elevation has not yet closed three structural gaps. Zimbabwe completed that self-assessment between 29 June and 4 July 2025, after the Plan had been drafted but before its launch, led by the Ministry of Health and Child Care with World Health Organization support and covering 19 technical areas [7]. An external validation mission followed from 6 to 12 September 2025, and its report had not been published when this piece was written [12]. The findings cited here are therefore self-assessed rather than externally validated. They still carry weight, because the Ministry rather than the Plan’s drafting team ran the exercise and reached much the same conclusions as the baseline assessment. Table 1 sets out the three gaps, the evidence for each and what each implies.

**Table 1.** Documented evidence on three gaps in the institutionalisation of Zimbabwe’s national One Health Strategic Plan 2026–2030, with the author’s interpretation of each.

Institutional gap	Documented evidence and source	Interpretation (author’s assessment)
Financing	Annex I of the Plan costs 30 activities at a total of US$11,483,468 for 2026–2030, which is an estimated implementation requirement for the full Plan period rather than a secured budget [1]. The Plan records that government meets salaries and infrastructure for the participating ministries but that there is ‘no specific budget allocation for specific One Health initiatives’ [1]. No amounts are apportioned between domestic and development-partner sources, and securing a government budget line is itself a costed advocacy activity of US$2,100 [1]. The baseline assessment concluded that sustainability ‘will require dedicated domestic funding’ [6]. Separately from the Plan, the Pandemic Fund allocated US$14,326,514.90 in February 2026 to a national pandemic preparedness project for 2026–2028 under its third round of funding, implemented through the African Development Bank, the Food and Agriculture Organization and the United Nations Children’s Fund, and launched in October 2026 [13]. That grant is external project financing channelled through those agencies, so it is neither an allocation to the Plan nor a line in the national budget.	The Plan is costed in full but not yet financed. Institutional survival is likely to depend on continued donor interest until a domestic allocation exists.
Legal mandate	The Plan lists 14 existing sectoral Acts relevant to One Health, so the gap is not a complete absence of legislation [1]. None of those Acts establishes the coordination mechanism itself, and the Plan’s own strategy is to review, harmonise and align existing laws, identify gaps and overlaps, and only then draft new legislation [1]. The 2025 self-assessment flagged ‘outdated or poorly implemented legal instruments’ and ‘limited availability of legal support at subnational levels’ [7]. The baseline assessment called for ‘[One Health] policy supported by legislation’ [6].	Coordination rests on executive goodwill rather than statute, and could weaken with a change in political leadership.
Subnational reach	The Plan’s text makes no reference to provincial, district or local-authority structures. The nearest reference is to advocacy at ‘national, regional, and community levels’ [1]. The 2025 self-assessment found ‘limited availability of legal support at subnational levels’ [7], and devolution to Zimbabwe’s three tiers of government remains legislatively and fiscally incomplete [8].	The Plan risks remaining a Harare-based initiative that never reaches the districts where health threats emerge.

Note: The second column reports findings documented in the cited sources. The third column is the author’s interpretation and is not a finding reported in any source.

## Weaknesses in cross-sectoral and laboratory systems

The same self-assessment found that coordination between the human, animal and environmental health sectors, the very sectors the Plan is meant to unify, remains weak, and that biosafety and biosecurity capacity, particularly laboratory infrastructure and staff training, needs strengthening [7]. Crisis response tends to favour visible coordinating structures over the less visible data systems on which joint action depends. Malawi offers a Southern African contrast, having built a One Health surveillance platform on its district health information infrastructure during the pandemic and sustained it afterward [14]. The Plan provides for an equivalent step, a national action plan for integrated One Health surveillance costed at US$217,500, listed as an activity still to be undertaken [1].

## Equity implications of incomplete institutionalisation

Plant-health agencies, including the Plant Quarantine Services Institute, took part in drafting the Plan, and its vision names plant health alongside human, animal and environmental health. Its surveillance priorities, however, centre on antimicrobial resistance and on zoonotic, neglected tropical and vector-borne diseases [1]. Three distinctions matter. First, plant-health risks with direct human and animal health consequences, such as fall armyworm, aflatoxin contamination of staple maize and pesticide residues in food chains, are not named anywhere in the Plan’s text, which is an absence from its stated priorities rather than evidence that they are neglected in practice. Second, whether plant health is built into operational surveillance cannot yet be assessed, because the action plan that would specify it has not been written. Third, the under-representation of plant health in One Health strategies is a documented global pattern rather than a Zimbabwean peculiarity [15]. My own reading is that a reform built around the threats most visible during the pandemic risks leaving smallholder livelihoods as exposed as before.

Two equity gaps are documented. Women perform an estimated 70–80% of agricultural labour in Zimbabwe but remain largely excluded from the land tenure, credit and extension services that determine resilience to shocks [16]. The 2025 self-assessment recorded gaps in funding for gender-based violence prevention and low representation of women in emergency preparedness and decision-making [7].

## What transfers and what does not

A recent systematic review of One Health operationalisation in Africa found limited resource availability and inadequate legal or institutional embedding to be recurring constraints. Its 13 included studies came predominantly from East, West and North Africa, however, and none examined a Southern African country [17]. A policy analysis of Botswana reports that the main barriers are organisational rather than technical, with fragmented responsibilities, poorly connected data systems and limited financing of joint work [18]. The 2025 Quadripartite joint statement for Africa calls for legislative support for national coordination mechanisms, institutional and workforce capacity and data systems, and urges domestic budget allocations alongside development-partner funding [19].

What travels is the sequencing question, whether financing, legal and subnational commitments are made alongside political elevation or deferred until after it. The arrangement itself does not, since presidential anchorage depends on a constitutional structure that gives the Office of the President and Cabinet an operational coordinating role, on the state of devolution, and on whether a standing One Health policy already exists, as it does in Rwanda but not yet in Zimbabwe [1,8,10]. Zimbabwe is best read as an illustrative case raising a broader question, not as a common regional trajectory.

## Conclusion

Three observations follow. Presidential coordination addressed a real, well-documented governance gap more ambitiously than most countries have. Yet the Plan, though fully costed, has no earmarked domestic allocation, no instrument establishing its coordination mechanism in law, and no reference to the provincial and district tiers that would implement it. Donor financing has arrived, but through international agencies rather than the national budget [13]. Political visibility has not yet converted into any of the three.

The recommendations are mine. Four entry points already exist in the Plan. The advocacy activity for a government budget line, costed at US$2,100, could become a Treasury vote at the next budget cycle. The legislative review could begin with a statutory instrument giving the One Health Secretariat terms of reference under existing Acts, not new legislation. The quarterly coordination meetings and technical working groups could turn a crisis mandate into routine cooperation, but only if attendance is specified at official as well as ministerial level and proceedings are reported to the Office of the President and Cabinet.

The integrated surveillance action plan is where responsibility could be assigned to a named subnational tier, which tier is unresolved because Zimbabwe’s devolution remains legislatively and fiscally incomplete [8]. Whether Zimbabwe takes these steps before 2030 will decide whether its pandemic-era innovation becomes an institution or a relic of crisis memory.

## Data Availability

Data sharing is not applicable to this article as no new data were created or analysed in this study.
